# The Development of a Coding Scheme for Intergenerational Learning and Its Application to the Patterns of Intergenerational Collaborative Communication

**DOI:** 10.3389/fpsyg.2021.629658

**Published:** 2021-02-16

**Authors:** Ya-Ling Wang

**Affiliations:** National Taiwan Normal University, Taipei City, Taiwan

**Keywords:** intergenerational learning, coding scheme, collaborative communication, game design, power process, communication skills, responses to bids

## Abstract

Much research has focused on intergenerational learning. However, its patterns and processes have rarely been explored. Therefore, this study aimed to develop a coding scheme for intergenerational learning, and to explore the patterns of collaborative communication emerging in the context of intergenerational learning. A total of 16 individuals (seven older adults and nine University students) participated in the study. Participants were invited to design digital games with their intergenerational team members. Of all the interactions, two sets of collaborative interactions were coded and analyzed. The findings revealed that the coding scheme for intergenerational learning was developed with high inter-rater reliability for three sub-systems: power process, communication skills, and responses to bids. Additionally, although the patterns of collaborative communication showed a balance of power during the task involving the collection of game elements, younger learners dominated during the task of completing the game prototype.

## Introduction

There has been accumulating attention paid and efforts made to investigate the advantages of having older and younger generations learn together across different countries (e.g., Boström, 2014; Franz and Scheunpflug, 2016; Spiteri, 2016; Gerpott et al., 2017; McKee and Scheffel, 2019; Sun et al., 2019; Yin et al., 2019; Santini et al., 2020). For example, Santini et al. (2020) conducted an intergenerational learning program in Germany, Italy, and Slovenia focusing on the field of organization and employment. During the program, the senior adults acted as mentors, and their entrepreneurial knowledge was valued for fostering the youngsters' entrepreneurial attitudes. However, little is known about how people from different generations communicate and contribute knowledge from their own specialties to learn together and collaborate (Strom and Strom, 2015). Therefore, this study aimed to develop and validate a coding scheme especially for collaborative communication during intergenerational learning; by doing so, three sub-systems in this coding scheme (power process, communication skills, and responses to bids) were created and qualified regarding their inter-rater reliability, and furthermore were validated by applying the current coding scheme in two different learning settings (one for the collection of game elements and one for completing a game prototype) to investigate and compare the collaborative communication patterns shown in two different contexts.

## Background

### Intergenerational Learning

“Intergenerational” is an adjective used to describe what actually occurs between different generations (Sánchez and Kaplan, 2014; Dauenhauer et al., 2016). Previous research investigating intergenerational relationships focused mainly on the reciprocity between parents and their children (e.g., Bó et al., 2020; Chai et al., 2020; Wang et al., 2020). Intergenerational learning is defined as the reciprocal exchange of knowledge between individuals from different generations. In doing so, people of all ages, whether relatives or strangers, are able to learn together and from each other (Sánchez and Kaplan, 2014). It should be noted that intergenerational learning emphasizes the centrality of exchange and reciprocity between different generations (Sánchez and Kaplan, 2014). In a multigenerational classroom, members of different generations possess some generational experience of belonging to different generations (Sánchez and Kaplan, 2014). Intergenerational learning not only refers to learning about others but also learning about oneself and one's own generational bearings (Sánchez and Kaplan, 2014).

#### Benefits of Intergenerational Learning

Intergenerational learning involves providing and designing learning and growth opportunities for learners across ages to deal with life and technological change. The impacts and benefits of intergenerational learning have been found to foster self-growth including active aging, self-esteem, and generativity (e.g., Giraudeau and Bailly, 2019; Lee et al., 2020). For example, Lee et al. (2020) conducted a scoping review to investigate senior adult-specific impacts and benefits that have been evaluated in intergenerational programs. Of the 28 coded studies, it was found that the impacts of joining intergenerational programs for older adults are related to the enhancement of ego integrity (Kim and Lee, 2017), positive affect (Marx et al., 2005; Baker et al., 2017; Kim and Lee, 2017), generativity (Scott et al., 2003; Ehlman et al., 2014; Andreoletti and Howard, 2018), self-confidence (McConnell and Naylor, 2016), and life satisfaction (Meshel and McGlynn, 2004; Gaggioli et al., 2014; DeMichelis et al., 2015).

In addition to the impact of intergenerational learning on the enhancement of older adults' self-growth, older adults, by joining intergenerational programs, are able to reconstruct their social networks and stay active with their family, community, and life (George et al., 2011; Gamliel and Gabay, 2014; Strom and Strom, 2015). Furthermore, it has been found that engaging in intergenerational learning may improve mutual understanding between generations, leading to enhanced positive feelings of acceptance and respect for people from various generations (George et al., 2011; Gamliel and Gabay, 2014; Tam, 2014).

However, previous research focused more on the outcomes and impacts of intergenerational learning. The process of how youngsters and older adults learn, interact, and even collaborate with each other during intergenerational learning has been explored less (Jarrott et al., 2008; Strom and Strom, 2015). Collaboration has been defined as each member being able to create a high degree of functionality in a team; that is, team members support each other to achieve their goals (De Schutter et al., 2017). Importantly, learning through intergenerational collaboration and communication, each with one's own generational awareness and experience, may well-engender intergenerational conflict and ambivalence (Sánchez and Kaplan, 2014). Additionally, some previous studies focused only on younger learners' outcomes, while others merely centered on the perspectives of the older learners (Dauenhauer et al., 2016). Research on intergenerational learning should focus on both younger and older learners' outcomes, and aim to understand the power process involved in meeting the standard of reciprocal exchange of knowledge (Sánchez and Kaplan, 2014).

Therefore, there is an urgent need to increase the understanding of intergenerational interaction, based on which mutual support between the different generations can be built. Thus, the current research aimed to develop a behavior coding scheme for both older and younger learners when they learn together, and to explore the patterns of collaborative communication during intergenerational learning.

### Coding Systems for Intergenerational Learning and Collaborative Interaction

Macroanalytic and microanalytic coding systems are different approaches to exploring behavior and interaction. The former have a global and gestalt-based focus, and are developed to investigate the major themes and bigger units of interaction. The latter are more appropriate when researchers aim to investigate smaller interaction units (Verstaen, 2017). Previous research on observing interaction among individuals mainly focused on couples and family members (e.g., Gottman and Driver, 2005; Darling et al., 2008; Friedlander et al., 2019). Studies on intergenerational partners and their interaction processes have, however, been accumulating because of the growing need for social structure (McKee and Scheffel, 2019; Sun et al., 2019; Yin et al., 2019).

For example, Rubin (2001) developed a play observation scale, which categorized play behaviors as *unoccupied* (e.g., inactivity)*, watching solitary* (e.g., activity without social interaction)*, parallel*, and *cooperative*. Also, Jarrott et al. (2008), modeling on Rubin's (2001) scale, developed an intergenerational observation scale (IOS) to investigate intergenerational interactions and affect. They expanded IOS to distinguish social targets such as age peers (e.g., interactive peers) and intergenerational partners (e.g., interactive intergenerational). In this scale, intergenerational behaviors are accordingly coded as seven categories: interactive intergenerational, parallel intergenerational, interactive peer, parallel peer, staff, watching solitary, and unoccupied.

To explore intergenerational interactions involving adolescents, institutionalized elderly, and older volunteers, Santini et al. (2018) developed two similar coding frameworks (one for older adults and one for adolescents). The categorization of adolescents' behaviors includes representation of older adults (e.g., bad/good, burden, experience exploitation, and useful), intergenerational relationship (e.g., conflictual, friendly, teach ICTs, understanding, listening, psychological support, tell, read, teach and mentor, and take care), lessons learnt from (awareness and changes of attitudes toward volunteering), and suggestions for improving the relationship with the youngest (the role of adults and the role of school authority). On the other hand, the categorization of older adults' behaviors involves the representation of young people (e.g., egoist, altruist, and able to listen), intergenerational relationship (e.g., indifference/distance, friendship, physical help, ICT literacy, company, love, and listening to each other), impact on (able to feel emotions and able to give love), and suggestions for improving the relationship with the youngest (more meeting chances, talking, systematic visits, more time spent together, and trips outside and fun).

According to the aforementioned coding systems, two key elements are extracted: behaviors and targets. Rubin (2001) used categorizations to represent a behavior without a specific target (e.g., solitary, parallel, and cooperative). For example, the categorization of cooperative means an individual doing something with a target; however, it still failed to refer to a specific target when doing something (i.e., age peer or intergenerational peer). Therefore, Jarrott et al. (2008) and Santini et al. (2018) employed categorizations involving one behavior and a specific target to represent with whom the individual does something. Nevertheless, few behavior coding systems have been customized and developed for collaborative communication during intergenerational learning. The current research accordingly aimed to develop a coding system for the assessment of collaborative communication regarding intergenerational learning.

### The Current Study: The Coding Scheme for Collaborative Communication During Intergenerational Learning

There is more to intergenerational learning models than just the participation of learners of different ages and generations. Diversity and enrichment could result not only from advancing age but also from various experiences, individual life events, and historical events (Whitehouse, 2017). To assess intergenerational group members' collaborative communication during intergenerational learning, the current study reviewed previous research especially focusing on the theme of intergenerational collaborative communication. According to previous research findings, the power process (McHale et al., 2015; De Schutter et al., 2017), communication skills (Suhr et al., 2004; De Choudhury and Kiciman, 2017), and the way of responding to bids (Driver and Gottman, 2004; Gable et al., 2006) are key factors of individual behavior and interaction among dyadic or group partners. Therefore, the current framework included these three core sub-systems, as described below.

#### Power Process

In previous research findings, older adults were more often found to serve as mentors to their younger counterparts (e.g., George et al., 2011; DeMichelis et al., 2015; De Schutter et al., 2017). They were not only able to encourage and build their younger partners' confidence, but could also lead them to perform behaviors which could meet social expectations (Tam, 2014). However, the balance of power structure and mutual contribution are key factors influencing the success of intergenerational learning. For example, De Schutter et al. (2017) conducted an intergenerational workshop named the Miami Six-O project. During the workshop, they analyzed how the balance of power was delivered and negotiated during the intergenerational interaction. They coded the process of each intergenerational interaction, and identified whether the senior adults or younger adults dominated, followed, disconnected from, or actively engaged in the collaboration. Their findings suggested that the discussions were dominated by either older or younger adults in half of the teams; the other half of the teams revealed collaborative interactions. Accordingly, to investigate whether both generations of intergenerational learners could contribute their own advantages, hold a balance of power, and even collaborate during intergenerational learning, the construct of power process was included in the current coding scheme.

#### Communication Skills

To examine how intergenerational learners learn and collaborate, assessing interactional communication is important (Strom and Strom, 2015). According to De Schutter et al. (2017), the construct of collaboration was defined as each member being able to mutually support each other to achieve the goals. Accordingly, social support is regarded as one of the crucial elements when team members perform collaborative communication. Additionally, previous research (e.g., Suhr et al., 2004) developed the social support behavior code for interaction. Subsequently, the construct of social support has been studied in a growing body of research investigating communication and interaction (e.g., Craig and Johnson, 2011; Bradford et al., 2012; Harel et al., 2012; Adams et al., 2014; De Choudhury and Kiciman, 2017). Therefore, the current framework includes the sub-scheme “communication skills” which evaluates how intergenerational learners communicate with and provide social support for each other.

#### Responses to Bids

Previous research (e.g., Driver and Gottman, 2004; Gottman and Driver, 2005) has indicated that responses to bids are important for understanding interpersonal daily interactions and for improving relationship quality. Responses to bids involve the ways of turning toward (acceptance and encouragement) and turning away from (ignoring or even disagreeing) one's partner when having a conversation (Driver and Gottman, 2004; Gottman and Driver, 2005). Additionally, researchers have also proposed the concept of *perceived responses to capitalization attempts* (Gable et al., 2006; Smith and Reis, 2012; PRCA). PRCA refers to a perception that individuals perceive their partners as being supportive following the disclosure of personal positive events involving four kinds of feedback: (1) active constructive (e.g., enthusiastic support); (2) passive constructive (e.g., quiet, understated support); (3) active destructive (e.g., quashing the event); and (4) passive destructive (e.g., ignoring the event). Accordingly, responses to bids and perceived responses are crucial for understanding conversation and daily interaction. The current coding scheme for intergenerational learning therefore includes the construct of responses to bids.

### Aims of the Current Research

The current research addressed the following research aims. First, the present research sought to develop a coding scheme for collaborative communication during intergenerational learning. Second, to establish the reliability of our coding scheme, we aimed to examine the inter-rater reliability of the coding scheme across coders. Third, to verify the validity and the application of the various learning conditions of intergenerational learning, the study aimed to explore how elderly adults and University students learn from each other, and how the balance of power is compromised and negotiated by creating two different collaborative tasks—one focusing more on the process of creation and the collection of the game elements and design, and the other placing more emphasis on the completion of an executable prototype. Finally, the current research aimed to investigate and compare two different collaborative communication patterns shown in two different learning contexts.

## Materials and Methods

### Participants and Designs

After developing the coding scheme for intergenerational learning, an intergenerational workshop was designed and held to investigate the reliability and application of this scheme, and to explore the communication patterns during intergenerational learning. A total of 16 individuals (seven older adults and nine University students) participated in this workshop. The average age of older adults was 62.33 years (SD=3.14) and the average age of University students was 21.22 years (SD=2.73). During the workshop, all the participants were invited to learn and design digital games with their intergenerational group members. They were required to design a game with two main purposes: (1) play and have fun with intergenerational partners; and (2) the game elements were to involve local culture elements (e.g., understanding festivals or community). Participants of two generations (i.e., older adults and University students) were, respectively, and randomly assigned to four groups; therefore, there was at least one older adult and one University student in each group. All participants provided written informed consent before participating in the workshop, and they were fully debriefed at the end. The participants in each group performing the two collaborative tasks are shown in Table 1.

**Table 1:** Participants in each group performing two collaborative tasks.

	**Participants**	**Task 1**	**Task 2**
G1	O11 (M)	V	
	O12 (F)	V	V
	Y11 (M)	V	V
	Y12 (M)		V
G1 total	4 (2O2Y)	3 (2O1Y)	3 (1O2Y)
G2	O21 (M)	V	V
	O22 (F)	V	V
	Y21 (F)	V	V
	Y22 (M)	V	V
G2 total	4 (2O2Y)	4 (2O2Y)	4 (2O2Y)
G3	O31 (F)	V	V
	O32 (M)	V	
	Y31 (F)	V	V
	Y32 (F)		V
G3 total	4 (2O2Y)	3 (2O1Y)	3 (1O2Y)
G4	O41 (F)	V	V
	Y41 (F)	V	V
	Y42 (F)	V	
	Y43 (F)	V	V
G4 total	4 (1O3Y)	4 (1O3Y)	3 (1O2Y)
Overall total	16 (7O9Y)	14 (7O7Y)	13 (5O8Y)

*M, male; F, female; O, older learner; Y, younger learner*.

### Introduction to the Intergenerational Game Design Workshop

The current workshop was modeled after the Miami Six-O project (De Schutter et al., 2017), and was tailored according to the collaborative tasks Previous research has investigated the benefits of games for elderly adults (e.g., McLaughlin et al., 2012). Also, digital game design was conducted in previous studies and was found to be a good way to enhance intergenerational relationships because technology has been proven to be a medium which changes the traditional dominant role of older adults into a more balanced and equal power in intergenerational relationships (e.g., Khoo et al., 2009; Al Mahmud et al., 2010; De Schutter et al., 2017; Cucinelli et al., 2018). Therefore, a workshop was accordingly created to explore and design digital games as a medium to facilitate intergenerational learning and intergenerational collaborative communication. Therefore, the aims of the workshop, as advertised to the University students and senior participants, was to learn together with intergenerational classmates to design a paper prototype of digital games for intergenerational players.

The workshop comprised a four-step creative process that was spread across 6 days over a 2-week period. Participants joined this workshop every 2 days during the 2-week workshop. The process was customized for intergenerational learning, and followed previous models (e.g., Howard et al., 2008; De Schutter et al., 2017). Based on the models of Howard et al. (2008) and De Schutter et al. (2017), the four stages were similar to the analysis, generation, evaluation, and implementation phases.

The instructors of this workshops are experts with game design and local culture backgrounds. They designed the courses of the workshop and led the participants based on the four stages (analysis, generation, evaluation, and implementation). Throughout the 2-week workshop, the intergenerational participants were instructed and guided through a structured process to analyze the current resources and conditions. In addition, individuals in each group were videotaped and recorded throughout the whole workshop.

#### Be a Chef! vs. Hey! Show Me Your Game!

The workshop involved four phases. Phase 1 (exploration of the similarities and differences of the generations) was designed to help intergenerational group members start to get to know each other. Phase 2 (creative brainstorming) aimed to generate ideas, develop concepts and develop confidence during the discussions. After Phase 1 and Phase 2, the intergenerational group members were required to integrate and implement their ideas and concepts collected from previous discussion as game designs (i.e., Phase 3: Be a chef!). Finally, the intergenerational participants were required to complete an executable prototype for exhibition (i.e., Phase 4: Hey! Show me your game!).

During the task “Be a chef!,” the intergenerational members were invited to provide game elements from their experience and knowledge without any frame limitation; in doing so, all “ingredients” were encouraged. The purpose of this task was to help the team members to form the concepts of games and to integrate and implement all “ingredients” into their game sketch. In “Hey! Show me your game!” the team members were required to complete an executable prototype based on the integrated design framework of game-based and playful learning (Plass et al., 2015). According to this framework, they were asked to complete a prototype with game elements involving knowledge/skills, an incentive system, learning and assessment mechanics, aesthetic design, narrative design, and a musical score.

### Coding Scheme and Data Analysis

The current coding system involving three sub-coding schemes (see below) was adopted for investigating intergenerational learning, accordingly, examining the first and second aims of the current research. The power process coding scheme included three codes and was adopted to investigate the third research aim. The communication skills coding scheme involved eight codes, and the response to bids coding scheme included seven codes. Three coding schemes were adopted to explore the fourth research aim.

#### Power Process

As shown in Table 2, the power process coding scheme was created for identifying the balance of power during intergenerational learning. It includes three interaction behavior codes, namely older learner in power (OP), younger learner in power (YP), and power balance (PB). OP was coded when the older learner was in power in the interaction unit of intergenerational learning; YP was identified when the younger learner was in power during the interaction learning; PB was coded when the two members displayed equal power during the intergenerational learning.

**Table 2:** Coding scheme for intergenerational learning.

**Code**	**Dimension/category**	**Construct measured**	**Definition**
OP	Older learner in power	Power process	Older learner voices insults, put-downs, demeaning remarks, threatening, manipulative statements or body language, overt commands or hostile demands to react, disagree, or even change one's partner's thoughts feelings, or actions
YP	Younger learner in power		Younger learner voices insults, put-downs, demeaning remarks, threatening, manipulative statements or body language, overt commands or hostile demands to react, disagree, or even change one's partner's thoughts feelings, or actions
PB	Power balance		Both older and younger learners show equality and a balance of power
OPS	Older learner with problem solving skills	Communication skills	Older learner displays constructive facilitation of discussion and problem solving
OW	Older learners' withdrawal		Older learner displays avoidance or distance regarding tone, body language, and attitude
OC	Older learner with conflict and negativity		Older learner displays anger, frustration, irritation, or blame
OO	Older learner being off-topic		Older learner discusses issues that are irrelevant to the topic
YPS	Younger learner with problem solving skills		Younger learner displays constructive facilitation of discussion and problem solving
YW	Younger learners' withdrawal		Younger learner displays avoidance or distance regarding tone, body language, and attitude
YC	Older learner with conflict and negativity		Younger learner displays anger, frustration, irritation, or blame
YO	Younger learner being off-topic		Younger learner discusses issues that are irrelevant to the topic
OTT	Older learner turning toward partner	Responses to bids	Older learner welcomes the bid and tries to respond
OTA	Older learner turning against partner		Older learner responds with contempt, belligerence, criticism, or defensiveness
OI	Older learner ignoring partner		Older learner shows lack of response to the bid by beginning a new bid or engaging in another activity
YTT	Younger learner turning toward partner		Younger learner welcomes the bid and tries to respond
YTA	Younger learner turning against partner		Younger learner responds with contempt, belligerence, criticism, or defensiveness
YI	Younger learner ignoring partner		Younger learner shows lack of response to the bid by beginning a new bid or engaging in another activity
O	Off-topic or others		The behavior unit is irrelevant to “responses,” or cannot be identified as one of the six abovementioned codes

#### Communication Skills

Communication skills refer to the behavior units showing positivity or negativity about the collaborative tasks during the intergenerational learning. As indicated in Table 2, the communication skills coding scheme consists of eight interaction behavior codes, namely older learner with problem solving skills (OPS), older learner's withdrawal (OW), older learner with conflict and negativity (OC), older learner being off-topic (OO), younger learner with problem solving skills (YPS), younger learner's withdrawal (YW), younger learner with conflict and negativity (YC), and younger learner being off-topic (YO).

#### Responses to Bids

Responses to bids is defined as the ways of responding to partners during intergenerational learning involving turning toward (i.e., acceptance and encouragement) and turning away from (i.e., ignoring or even disagreeing with) one's partner. As shown in Table 2, the responses to bids coding scheme includes seven codes: older learner turning toward partner (OTT), older learner turning against partner (OTA), older learner ignoring partner (OI), younger learner turning toward partner (YTT), younger learner turning against partner (YTA), younger learner ignoring partner (YI), and off-topic or others (O).

### Quantitative Content Analysis

For the quantitative content analysis, since Phases 1 and 2 mainly focused on cultivating team camaraderie and exploring familiarity among team members, the intergenerational participants only started to get to know their group members and had not yet developed enough familiarity and confidence during the discussions. Accordingly, the current study aimed to analyze the patterns of discussion in Phases 3 and 4. In view of this, the researchers only watched and analyzed the videos of “Be a chef!” and “Hey! Show me your game!”

In addition, to investigate the inter-rater reliability of the current coding scheme for intergenerational learning and the exploration of patterns of collaborative communication during different tasks, we chose two 20-min collaborative interactions (one from Phase 3 and one from Phase 4) to code and analyze the intergenerational learning. The video data of one sampled intergenerational interaction were first coded by two well-trained coders with education or psychology backgrounds.

**Figure 1:**
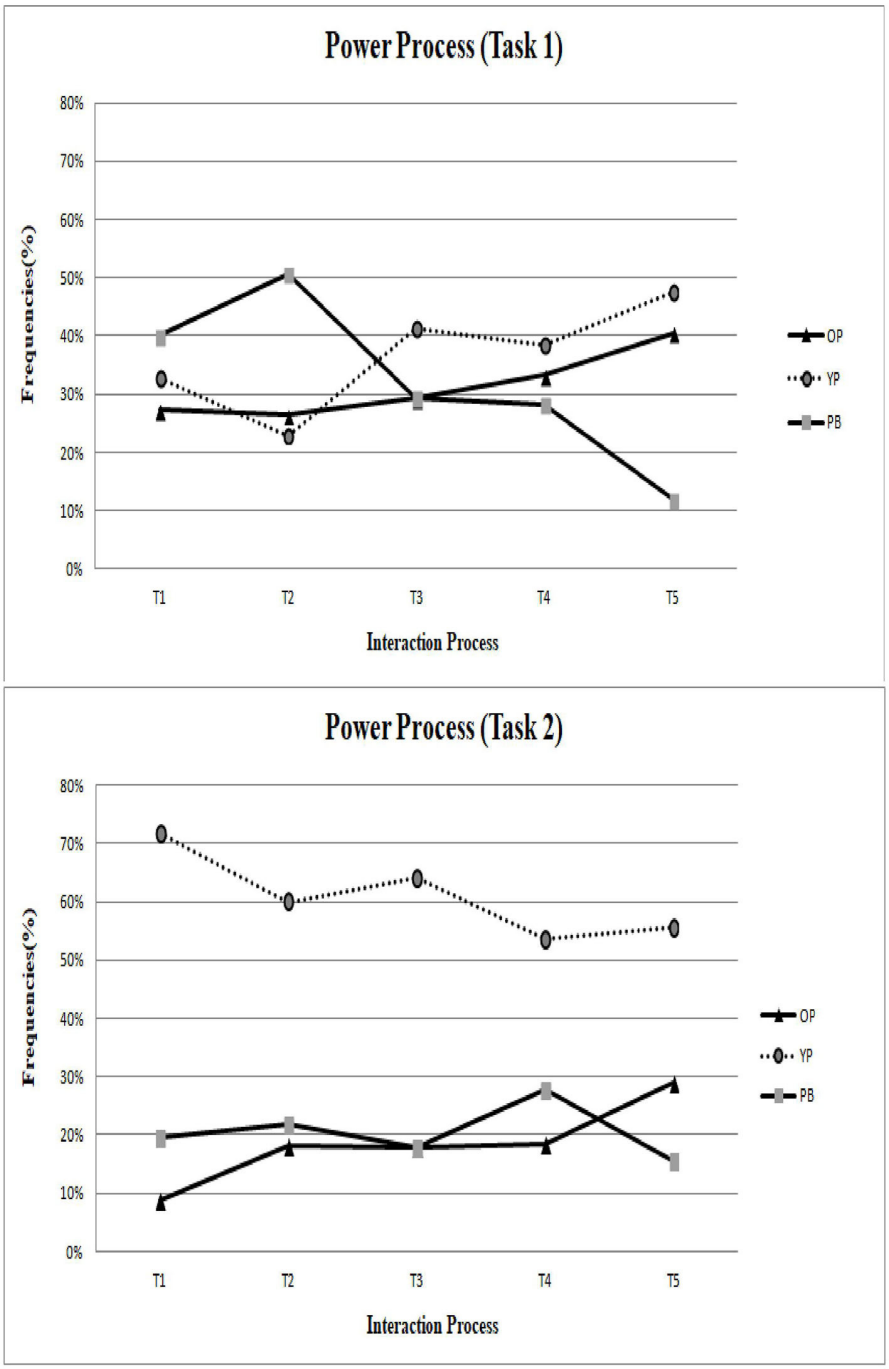
The patterns of power process in Task 1 and Task 2.

**Figure 2:**
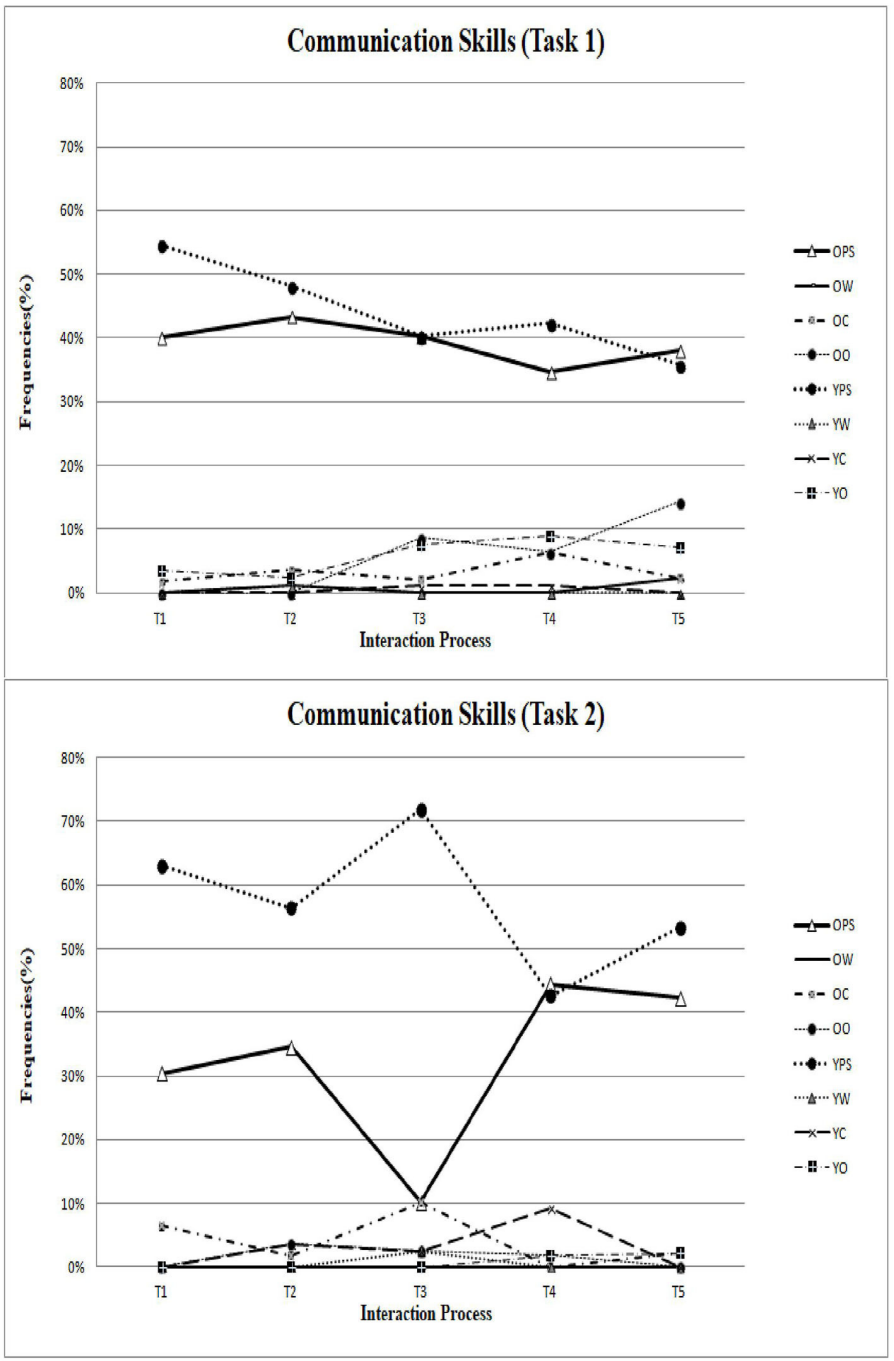
The patterns of communications skills in Task 1 and Task 2.

**Figure 3:**
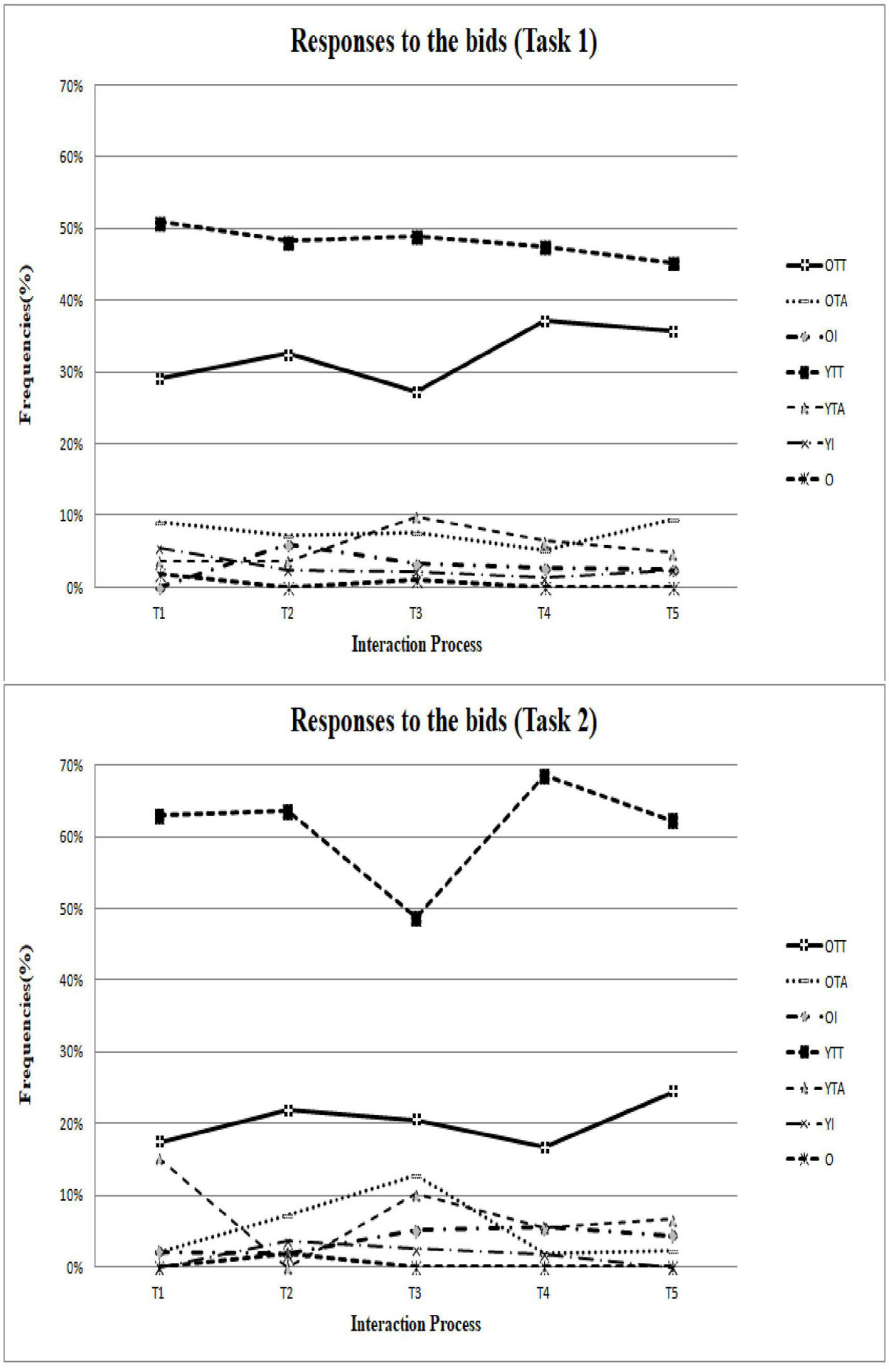
The patterns of response to the bids in Task 1 and Task 2.

After determining the inter-rater reliability, all video data of collaborative interaction in Task 1 (Be a chef!) were divided into a total of 350 interactional collaborative behavior units across the four groups. Additionally, data of the collaborative interaction in Task 2 (Hey! Show me your game!) were divided into a total of 239 interactional collaborative behavior units across the four groups. Furthermore, to better understand the sequential process of intergenerational discussion, the current research also divided 20-minute collaborative interactions into five intervals (refer to Figures 1–**3**) to review the change in patterns during Tasks 1 and 2.

## Results and Discussion

### Inter-rater Reliability and Frequency

Results of Kappa coefficient *k* revealed the high inter-rater reliability of the codes (Landis and Koch, 1977) for the power process (*k* = 0.87; *p* < 0.001), communication skills (*k* = 0.91; *p* < 0.001), and responses to bids (*k* = 0.85; *p* < 0.001). Table 3 shows the frequencies and percentages of each interaction code during the discussions in Task 1 and Task 2 for each group, individually and across the four groups. The overall findings revealed that, in Task 1, almost one third of the interaction codes show that the older adults were in charge of the discussion; slightly more than one third show that the younger participants played a leading role during the discussion; and one third show the balance of power structure during the discussion. Our patterns tended to be consistent with the findings of De Schutter et al. (2017), showing that the discussions were dominated by either older or younger adults in half of the teams, while the other half of the teams revealed collaborative interactions. The patterns of Task 2 are, however, completely different from those of Task 1, showing that the younger participants in the discussion regarding an executable game prototype guided and led more than their senior counterparts.

**Table 3:** Frequencies and percentages of each interaction code during the discussions.

	**G1**				**G2**				**G3**				**G4**				**Overall**			
	**Task 1**		**Task 2**		**Task 1**		**Task 2**		**Task 1**		**Task 2**		**Task 1**		**Task 2**		**Task 1**		**Task 2**	
	**Freq.**	**%**	**Freq**	**%**	**Freq**	**%**	**Freq**	**%**	**Freq**	**%**	**Freq**	**%**	**Freq**	**%**	**Freq**	**%**	**Freq**	**%**	**Freq.**	**%**
**POWER PROCESS**																				
OP	49	43.8%	0	0.0%	25	33.3%	33	44.00%	30	42.9%	10	26.3%	3	3.2%	1		107	30.6%	44	18.41%
YP	41	36.6%	59	96.7%	23	30.7%	23	30.67%	25	35.7%	17	44.7%	59	63.4%	46	70.8%	148	42.3%	145	60.67%
PB	22	19.6%	2	3.3%	27	36.0%	19	25.33%	15	21.4%	11	28.9%	31	33.3%	18	27.7%	95	27.1%	50	20.92%
Total	112	100.0%	61	100.0%	75	100.0%	75	100.00%	70	100.0%	38	100.0%	93	100.0%	65	100.0%	350	100.0%	239	100.00%
**COMMUNICATION SKILLS**																				
OPS	50	44.6%	1	1.6%	29	38.7%	38	50.67%	41	58.6%	21	55.3%	18	19.4%	20	30.8%	138	39.4%	80	33.47%
OW	1	0.9%	0	0.0%	0	0.0%	0	0.00%	1	1.4%	0	0.0%	0	0.0%	0	0.0%	2	0.6%	0	0.00%
OC	4	3.6%	1	1.6%	4	5.3%	8	10.67%	4	5.7%	0	0.0%	0	0.0%	0	0.0%	12	3.4%	9	3.77%
OO	4	3.6%	0	0.0%	5	6.7%	0	0.00%	2	2.9%	0	0.0%	8	8.6%	4	6.2%	19	5.4%	4	1.67%
YPS	53	47.3%	58	95.1%	36	48.0%	22	29.33%	21	30.0%	15	39.5%	45	48.4%	40	61.5%	155	44.3%	135	56.49%
YW	0	0.0%	0	0.0%	0	0.0%	0	0.00%	0	0.0%	1	2.6%	1	1.1%	0	0.0%	1	0.3%	1	0.42%
YC	0	0.0%	1	1.6%	1	1.3%	7	9.33%	1	1.4%	0	0.0%	0	0.0%	0	0.0%	2	0.6%	8	3.35%
YO	0	0.0%	0	0.0%	0	0.0%	0	0.00%	0	0.0%	1	2.6%	21	22.6%	1	1.5%	21	6.0%	2	0.84%
Total	112	100.0%	61	100.0%	75	100.0%	75	100.00%	70	100.0%	38	100.0%	93	100.0%	65	100.0%	350	100.0%	239	100.00%
**RESPONSES TO THE BIDS**																				
OTT	45	40.2%	1	1.6%	32	42.7%	31	41.33%	18	25.7%	7	18.4%	17	18.3%	9	13.8%	112	32.0%	48	20.08%
OTA	9	8.0%	0	0.0%	7	9.3%	12	16.00%	8	11.4%	0	0.0%	2	2.2%	0	0.0%	26	7.4%	12	5.02%
OI	6	5.4%	1	1.6%	2	2.7%	3	4.00%	3	4.3%	4	10.5%	0	0.0%	1	1.5%	11	3.1%	9	3.77%
YTT	46	41.1%	55	90.2%	26	34.7%	21	28.00%	38	54.3%	26	68.4%	59	63.4%	46	70.8%	169	48.3%	148	61.92%
YTA	5	4.5%	3	4.9%	4	5.3%	8	10.67%	3	4.3%	0	0.0%	9	9.7%	6	9.2%	21	6.0%	17	7.11%
YI	1	0.9%	0	0.0%	2	2.7%	0	0.00%	0	0.0%	1	2.6%	6	6.5%	3	4.6%	9	2.6%	4	1.67%
O	0	0.0%	1	1.6%	2	2.7%	0	0.00%	0	0.0%	0	0.0%	0	0.0%	0	0.0%	2	0.6%	1	0.42%
Total	112	100.0%	61	100.0%	75	100.0%	75	100.00%	70	100.0%	38	100.0%	93	100.0%	65	100.0%	350	100.0%	239	100.00%

### The Collaborative Patterns During the “Be a Chef!” Task

To meet the third aim of the current research, the study created two tasks with different purposes to explore how elderly adults and University students learn from each other, and how the balance of power is compromised and negotiated during intergenerational learning. As shown in the upper part of Figures 1, 2, both senior and younger participants displayed equality and contributed mutually to the coding scheme of the power process and communication skills.

The patterns indicated egalitarian intergenerational collaboration consistent with previous research findings. For example, according to the research findings of DeMichelis et al. (2015), both older and younger participants actively engaged in the activities. Additionally, intergenerational learning serves as a reciprocal interaction whereby both older and younger generations show unique contributions (Borrero, 2015). However, each generation demonstrated different behavioral patterns in various ways. For instance, older adults tended to offer younger partners real-world understandings of concepts, and were good at mentally reflecting themselves backwards and forwards across different life experiences; conversely, younger counterparts were more adept at providing educational knowledge and learning from others' life courses (Borrero, 2015; DeMichelis et al., 2015). Additionally, according to a study on the healing effects of intergenerational dialogue, the results revealed that the elders were willing to share personal and positively helpful stories from their history and experiences, whereas the youth were eager to learn from older counterparts' past experiences (Wallace et al., 2014).

### The Collaborative Patterns During the “Hey! Show Me Your Game!” Task

As shown in the lower part of Figures 1–3, the younger participants played a dominating role in the coding schemes of power process, communication skills, and responses to bids. The patterns indicated that younger partners are more capable of providing their educational experience and their knowledge of technology to their older partners, consistent with previous research findings.

Derboven et al. (2012) conducted a study regarding intergenerational collaboration. Participants were invited to join an intergenerational user interface discussion and game evaluation. The findings revealed that younger participants put more effort and time into guiding and helping their older partners on the user interface. It was also suggested that younger players enjoyed teaching and explaining how to play games to their older partners. Younger partners are more capable of sharing their experience with and knowledge of technology with their older partners (Derboven et al., 2012).

Additionally, Alfrey et al. (2020) suggested that the traditional power structure within intergenerational relationships may be disturbed and overcome if youth are given the opportunity to play the role of teacher to their older counterparts. For example, it was suggested that opportunities be created for younger participants to teach older participants how to use mobile phones.

Overall, the current research makes a unique contribution to intergenerational learning. It also provides implications for future studies and educators as follows. First, our findings developed a coding scheme for intergenerational learning, and illustrated two patterns of collaboration when tasks with different purposes were delivered. Second, the validation of the coding scheme for intergenerational learning showed a reciprocal and mutual contribution during the task regarding the process of creation and collection of the game elements and design. The findings were consistent with previous research results. For instance, Alfrey et al. (2020) conducted intergenerational arts-led pedagogies, and their findings suggested that the intergenerational arts-led pedagogies worked in connecting different generations. Additionally, through pedagogies, stereotypes of the other generation and the power structure between the generations were disturbed and shifted.

Nevertheless, a previous study indicated that egalitarian intergenerational collaboration requires great energy and effort due to the age-based inequality (Taft, 2015). Intergenerational dialogue, despite many efforts to foster youth's authority and agency, continues to exhibit deeply structured patterns of interaction that give elders greater power. However, the current findings successfully showed a technique (i.e., teaching how to design a digital game) to disturb the traditional power structure of intergenerational collaboration. The findings paralleled the results of Guha et al. (2013), indicating that the power issues in intergenerational relationships exist, but that these issues can be resolved. To change the power structure in intergenerational relationships, numerous techniques were used in their study to overcome the power differentials. For example, they arranged intergenerational participants to have fun together by playing outside, sitting together on the floor, and visiting a campus.

Dauenhauer et al. (2016) employed an intergenerational approach to investigate the promotion of balance and strength for fall prevention. The findings revealed that intergenerational intervention is effective in terms of the prevention of fall risk in seniors and younger participants. It was also suggested that future research should pay more attention to the issues of whether intergenerational programs are equal to every generation or serve as age-specific programs (Granacher et al., 2011; Alfrey et al., 2020).

Last but not least, the current findings should be interpreted with caution. We list the limitations with corresponding future research directions as follows. First, the current research investigated participants using the observation method, which is a research technique where researchers observe individuals' ongoing behavior in a natural situation. Accordingly, its conclusions should be limited to this particular collaborative task and a limited number of participants. Future research may collect data using other methods such as experimental tasks, questionnaire surveys, or employing a longitudinal design. Future research may sequentially investigate intergenerational learning and intergenerational collaborative interaction from different waves of observation or investigations, thereby establishing the causal relationships. Future studies could incorporate more investigations in various situations to explore intergenerational learning in greater depth. Second, the collaborative patterns shown in Figures 1–3 were compared based on the five intervals of 20-min interactions, which failed to present significantly sequential behavioral patterns. To better understand the behavioral sequential patterns, future research could consider choosing sequential analysis (Bakeman and Gottman, 1997; Hou, 2010, 2015; Cheng and Hou, 2015; Wu et al., 2016), which is another type of analysis for behavior and interaction. During sequential analysis, the coded data of behavior codes are used to ascertain sequential behavior patterns. Nevertheless, this idea is speculative and should be investigated in future research.

## Conclusion

In exploring what this current study suggests about intergenerational learning, the findings have developed a coding scheme for intergenerational learning with high inter-rater reliability for three sub-systems: power process, communication skills, and responses to bids. In addition, the current research designed two tasks with different purposes, and further compared collaborative patterns during these two tasks. Although the patterns of collaborative communication showed a balance of power during the task regarding the collection of game elements (i.e., the “Be a chef!” task), younger learners dominated during the task of completing the game prototype (i.e., the “Hey! Show me your game!” task). The patterns indicated that younger partners are more capable of providing their educational experience and their knowledge of technology to their older partners.

## Data Availability Statement

The raw data supporting the conclusions of this article will be made available by the authors, without undue reservation.

## Ethics Statement

The studies involving human participants were reviewed and approved by National Taiwan Normal University. The patients/participants provided their written informed consent to participate in this study.

## Author Contributions

The author confirms being the sole contributor of this work and has approved it for publication.

## Conflict of Interest

The author declares that the research was conducted in the absence of any commercial or financial relationships that could be construed as a potential conflict of interest.
